# Inhibition of Influenza A Virus by Human Infant Saliva

**DOI:** 10.3390/v11080766

**Published:** 2019-08-20

**Authors:** Brad Gilbertson, Kathryn Edenborough, Jodie McVernon, Lorena E. Brown

**Affiliations:** 1 Department of Microbiology and Immunology, The University of Melbourne at the Peter Doherty Institute for Infection and Immunity, Melbourne, Victoria 3000, Australia; 2 Victorian Infectious Diseases Reference Laboratory Epidemiology Unit at the Peter Doherty Institute for Infection and Immunity, The University of Melbourne and the Royal Melbourne Hospital, Melbourne, Victoria 3000, Australia

**Keywords:** influenza virus, saliva, innate inhibitors, human infant, mouse, ferret

## Abstract

Innate antiviral factors in saliva play a role in protection against respiratory infections. We tested the anti-influenza virus activities of saliva samples taken from human infants, 1–12 months old, with no history of prior exposure to influenza. In contrast to the inhibitory activity we observed in mouse and ferret saliva, the activity of human infant saliva was complex, with both sialic acid-dependent and independent components, the proportion of which differed between individuals. Taken as a whole, we showed that the major anti-influenza activity of infant saliva is acquired over the first year of life and is associated with sialic acid-containing molecules. The activity of sialic acid-independent inhibitors was lower overall, more variable between individuals, and less dependent on age. The results show that the saliva of very young infants can provide a degree of protection against influenza, which may be critical in the absence of adaptive immunity.

## 1. Introduction

The influenza A virus (IAV) is an important respiratory pathogen and a major cause of global morbidity and mortality. Innate immunity is critical in the early stages of IAV infection, limiting the escalation of viral loads in the respiratory tract prior to the induction of the adaptive immune response. A variety of soluble innate inhibitors present in respiratory secretions participate in the early containment of IAV including collectins (surfactant protein D (SP-D), SP-A, and mannose binding protein (MBP)), pentraxins (long PTX3 and short SAP), mucins, salivary scavenger receptor cysteine-rich glycoprotein-340 (gp-340), and defensins [1,2,3,4,5,6,7,8,9]. These molecules interact with the virus and inhibit its function using one of two mechanisms. Conglutinin, SP-D, MBL, and SAP act as classic β-inhibitors, neutralizing virus infectivity through binding of their lectin domains to carbohydrate side chains located on the head of the influenza hemagglutinin (HA) of susceptible strains [8,10,11]. These molecules sterically block access of the HA to cell surface receptors [2,9]. SP-A, gp-340, mucins, PTX3, and α-2-macroglobulin (A2M) act as classic γ-inhibitors, mediating the inhibition of IAV by binding the viral HA to terminal sialic acid expressed by the molecule [12,13,14]. These inhibit influenza virus by providing decoy sialic acid ligands to which the virus binds [5,15]. In general, these soluble innate inhibitors and their mechanisms of action are well conserved in different animal species.

A subset of these innate inhibitors including A2M, MUC5B, and gp-340 has been found in saliva and may contribute to the first line of defense against influenza [5,16,17,18,19,20] and other viruses including human immunodeficiency virus type 1 [17,21] and herpes simplex virus [22]. In addition, we recently described an inhibitor of IAV in mouse saliva with a mechanism of action not analogous to any of the β- or γ-inhibitors described previously [23,24]. This inhibitor reduced the effectiveness of IAV replication by specifically targeting the viral neuraminidase (NA) and severely limited the ability of the virus to spread from the upper respiratory tract to the tracheas and lungs of infected animals. Inhibition by mouse saliva was shown to be independent of treatment with bacterial sialidase, receptor-destroying enzyme (RDE), implying that the mechanism of inhibition did not involve sialic acid. Whether a similar inhibitor is also naturally produced in other species including humans is not known. If such an inhibitor exists, it could provide very potent early protection of the lower respiratory tract against influenza infection prior to the development of acquired immunity. 

Previous reports in the literature describing binding to or inhibition of IAV and other viruses by human saliva have used samples taken from children, adult donors, or donors of unspecified age [5,16,18,19,20,21,22,25,26,27,28,29,30,31,32]. A potential confounder of these studies is that the donors have likely been previously exposed to influenza, either by natural infection or vaccination. Although Hartshorn et al. [5] have previously noted that the removal of IgA only slightly reduced the antiviral activity of saliva, the question remains whether the presence of specific antibodies to the influenza virus have the potential to modulate the activity of certain innate inhibitors, thus complicating the interpretation of the results. It is also not known whether prior exposure to the influenza virus can change the relative abundance of innate inhibitors present in mucosal secretions. As far as we can ascertain, there have been no attempts to investigate the inhibitory properties of infant saliva taken before virus exposure, hence before the acquisition of any acquired immunity. In this study, we sought to recruit newborn infants (<12 months old) with no prior exposure history of influenza to investigate whether their saliva contained innate inhibitors with activity against the virus and if so, were their mechanisms of action analogous to those previously described for IAV.

## 2. Materials and Methods

### 2.1. Cells and Viruses

Madin-Darby canine kidney (MDCK) cells were maintained in Roswell Park Memorial Institute (RPMI)-1640 media (Sigma-Aldrich, Castle Hill, Australia), supplemented with 10% heat-inactivated fetal calf serum (Life Technologies, Mulgrave, Australia), 2 mM l-glutamine (Sigma-Aldrich), 2 mM sodium pyruvate (Thermo Fisher, Scoresby, Australia), 24 μg/mL gentamicin (Pfizer, West Ryde, New South Wales, Australia), 50 μg/mL streptomycin (Life Technologies), and 50 IU/mL penicillin (Life Technologies). Media as above without serum were used for dilutions and the same medium containing 1 mg/mL of bovine serum albumin (BSA) (RPMI + BSA) was used as a control for the virus neutralization assays. All media were sterilized by membrane filtration with a pore size of 0.45 μM and stored at 4 °C. Cells were maintained at 37 °C in 5% CO_2_. 

Influenza strains A/Puerto Rico/8/34 (H1N1), A/Udorn/307/72 (H3N2), and A/California/7/09 (H1N1) were propagated in 10-day old embryonated hens’ eggs at 35 °C for 2 days before the allantoic fluid was harvested and stored at −80 °C. A/Wisconsin/15/09 (H3N2) and B/Brisbane/60/08 were provided by CSL Ltd. (Parkville, Victoria, Australia).

### 2.2. Collection of Saliva

Twenty-three saliva samples from nineteen infants were collected by parents supplied with disposable transfer pipettes and sterile 5 mL tubes. Whole saliva samples were centrifuged at 13,000 rpm for 10 min at 4 °C to remove particulates. The supernatant was collected, and penicillin and streptomycin added at a final concentration of 50 IU/mL and 50 μg/mL, respectively, to inhibit bacterial growth. Samples were stored frozen at −80 °C until used. The collection of samples was by informed consent of the parents and approved by the University of Melbourne Ethics Committee (Project #1034799). 

To obtain mouse saliva, mice were anesthetized using isofluorane and injected i.p with 200 μL of 20 μg/mL Carbachol (Carbamycholine Chloride, Sigma, MO, USA) in PBS and the saliva collected. To obtain ferret saliva, male ferrets were sedated with xylazine and injected with 700 μL of Carbachol as above. Saliva (0.5–1.0 mL) and sera were collected from individual ferrets, which were confirmed to have no prior exposure to Udorn and PR8 viruses in the hemagglutination inhibition assays.

### 2.3. Treatment of Saliva with Receptor-Destroying Enzyme (RDE)

Where specified, saliva was treated with RDE to remove sialic acid. One volume of saliva in 8 volumes of Ca-Mg saline (0.24 mM CaCl_2_, 0.8 mM MgCl_2_, 20 mM boric acid, 0.14 mM sodium tetraborate, 0.15 M NaCl) was treated with one volume of *Vibrio cholerae* RDE (Sigma, MO, USA) for 30 min at 37 °C. The final concentration of RDE was 50 mU/mL. RDE was then inactivated by incubation at 56 °C for 1 h. Samples were transferred to 1000 MW cut-off Spectrapor membrane tubing and dialyzed against 50 mM NH_4_HCO_3_ at 4 °C overnight. Following freeze-drying, samples were reconstituted to their original volumes with PBS and stored at −20 °C. 

### 2.4. Virus Neutralization Assay

Plaque reduction on MDCK monolayers cultured in TC6 plates was used to measure the neutralization of virus infectivity. Dilutions of virus were prepared in RPMI to give approximately 5000 plaque-forming units (pfu) per 10 μL and then mixed with untreated or RDE-treated saliva at a volume to volume (*v*/*v*) ratio of 1 virus:9 saliva. Mixtures of virus with RPMI + BSA (1 mg/mL) at the same ratio was used as a control. The mixtures were then incubated at 37 °C for 30 min before being added to MDCK cells, which were then overlaid with an agarose-medium mixture and infectious virus enumerated three days later by the ability to form plaques as described previously [33]. Virus neutralization was expressed as the percentage reduction in titer of virus obtained by pre-incubation with saliva compared to that recovered after incubation with RPMI + BSA. 

### 2.5. Hemagglutination Inhibition (HI) Assay 

Tests were performed in round-bottom 96-well microtiter plates at room temperature using 1% *v*/*v* chicken erythrocytes. Hemagglutination titers were determined by the titration of virus samples in PBS followed by the addition of an equivalent volume of erythrocytes. For hemagglutination inhibition tests, dilutions of RDE-treated saliva were prepared in PBS and four hemagglutinating units (HAU) of virus were added. Following 30 min of incubation, chicken erythrocytes were added and the ability of saliva to inhibit virus-induced hemagglutination was assessed after 30 min.

### 2.6. Statistical Analyses

Statistical analyses were determined with GraphPad Prism version 8.2.0 using a one-way ANOVA with Tukey’s multiple comparison test. Statistical significance was demonstrated by a *p* value < 0.05. Seemingly unrelated regression analyses were performed using Stata version 13.1 to compare coefficients (with 95% confidence intervals) describing the relationship between salivary virus inhibition and age over the first year of life for different viruses, without and with RDE treatment. Each individual was represented only once in each analysis, with the exception of the age analysis (violin plot), where multiple samples from three individuals at different ages were also included.

## 3. Results and Discussion

### 3.1. Infant Saliva Is Largely Devoid of Antibodies to Current and Historic Influenza Strains

Saliva samples (*n* = 23) were collected by parents from their infants (*n* = 19; males = 10, females = 9) who ranged in age from 7.0 to 51.9 weeks (mean = 28.5 weeks, median = 28.7 weeks) (Table A1). Female infants tended to be younger (mean 23.8 weeks, median 20.6 weeks) than males (mean 32.7 weeks, median 36.5 weeks), but this difference was not statistically significant, likely due to small group sizes. Factors such as the time of sample collection relative to the influenza season are not expected to influence the abundance or properties of inhibitors in saliva. It should be noted that, while recruitment into the study was not difficult, a number of parents consented to take part but ended up not providing a sample or the sample volume was extremely small, possibly due to the challenging nature of extracting saliva from infants (despite drooling). As such, receipt of a limited volume of some samples meant that not all tests could be performed for some donors.

**Figure 1:**
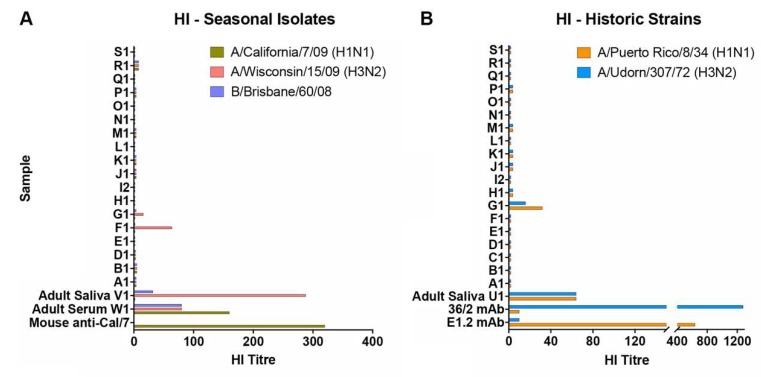
Hemagglutination inhibition activity of human infant saliva. The ability of saliva samples to inhibit virus-induced hemagglutination of chicken erythrocytes was assessed against (**A**) A/California/7/09 (H1N1), A/Wisconsin/15/09 (H3N2), and B/Brisbane/60/08 viruses and (**B**) PR8 (H1N1) and Udorn (H3N2). Adult serum and saliva samples, mouse antiserum raised against inactivated purified A/California/7/09 (a gift from CSL Ltd.), and HA-specific monoclonal antibodies against PR8 (E1.2) or Udorn (36/2) were included as controls. Data represent the mean of at least duplicate samples.

To determine whether donors had previously been exposed to influenza, the panel of saliva samples was initially tested for hemagglutination inhibition (HI) against A/California/7/2009 (H1N1), A/Wisconsin/15/2009 (H3N2), and B/Brisbane/60/2008 influenza viruses, strains antigenically related to those circulating in the human population at the time of sample collection (Figure 1A). Specificity and sensitivity of the assay was validated using saliva and serum samples taken from adult donors and mouse antibodies. High HI titers (80–160) against the three strains could be detected in the adult serum of W1 and the saliva from V1 showed a high titer against the H3N2 virus, a lower titer against the influenza B strain, and no response to the H1N1 virus. None of the infant saliva samples had detectable HI titers against the H1N1 or B virus strains and only one of the samples (F1) had a positive titer (HI = 64) against A/Wisconsin/15/2009, suggesting prior H3N2 infection. This result was consistent with a previous report from the mother that F1 had suffered from an influenza-like respiratory illness (ILI) around the time of recruitment into the study.

The dominant murine salivary inhibitor was previously characterized by its broad reactivity against influenza strains with the A/Udorn/307/72 (Udorn; H3N2) virus being one of only three seasonal strains identified that were relatively resistant to inhibition [23]. In contrast, A/Puerto Rico/8/34 (PR8; H1N1), which is highly virulent for mice when delivered to the lung bypassing saliva, was extremely sensitive to the NA-targeted inhibitor when the virus was delivered to the upper respiratory tract [23]. For this study, we used these two historic strains to investigate the inhibitory properties of human infant saliva, first to enable us to identify “mouse-like” inhibition, and second because infants could not have been previously exposed to these viruses and are unlikely to have induced antibodies that could potentially confound the results of subsequent neutralization assays. To confirm the latter, infant saliva was also tested for HI against the PR8 and Udorn viruses. As expected, the majority of the infant saliva samples had undetectable HI titers to these strains (Figure 1B) including F1. Sample G1 had detectable but low titers.

### 3.2. Infant Saliva Showed a Complex Pattern of Inhibition Unlike That Seen in the Mouse or Ferret

**Figure 2:**
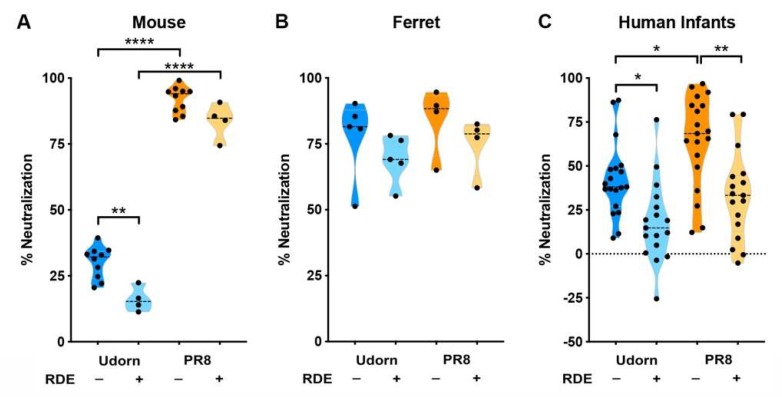
Inhibition of IAV by saliva of different species. PR8 or Udorn virus (5000 PFU) were added to untreated or RDE-treated (**A**) mouse (**B**) ferret or (**C**) human infant saliva at a virus/saliva ratio of 1:9 (vol/vol) for 30 min at 37 °C and the amount of infectious virus recovered was determined by a plaque assay on MDCK cells. The virus recovered in the presence of saliva was expressed as a percentage of that recovered when saliva was mixed with the mock-treated control. Violin plots indicating the mean of each group and probability density of the data at different values are shown. Data are representative of 4–10 independent experiments with pooled mouse saliva or at least three replicate individual saliva samples taken from 4–5 ferrets or 17–19 human infants.

Untreated and RDE-treated saliva samples were then tested for their ability to neutralize PR8 and Udorn in a plaque neutralization assay (Figure 2) and the pattern of inhibition of the infant saliva compared to that previously described in mice [23]. Murine saliva caused a relatively low level of inhibition of Udorn that was predominantly RDE-sensitive (*p* < 0.01 compared to untreated saliva) (Figure 2A). In contrast, PR8 was highly neutralized by mouse saliva (*p* < 0.0001 compared to Udorn) and inhibition was RDE-resistant (no difference between RDE-treated and untreated saliva). The ferret is another widely used model to study influenza virus pathogenesis, so we also tested whether ferret saliva showed a comparable inhibitor profile. As shown in Figure 2B, the pattern of inhibition in the ferret was vastly different from that of the mouse. Ferret saliva neutralized both strains to an equivalently high level and inhibition of both strains was RDE-resistant.

**Figure 3:**
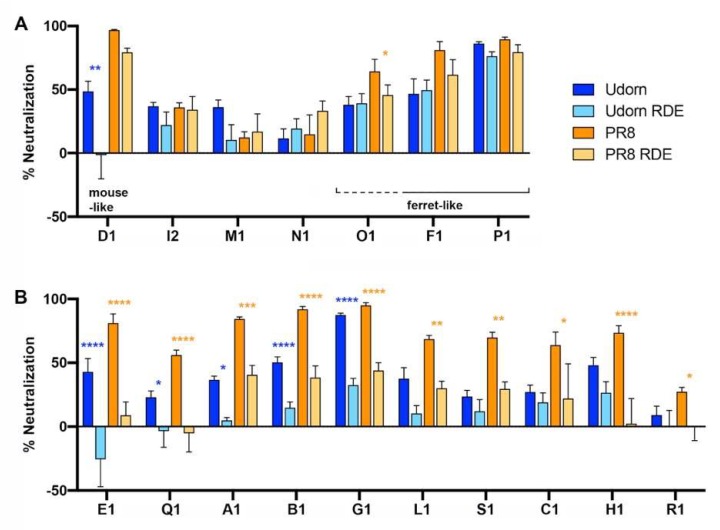
Inhibition of IAV by individual infant saliva samples. PR8 or Udorn virus (5000 PFU) were added to untreated or RDE-treated human infant saliva as described for Figure 2. The data represent the percentages of virus neutralized by saliva compared to control mixtures containing 5000 PFU of virus and RPMI + BSA. The means and standard deviations of at least three replicate samples are shown and represent data obtained from 17 individual human infants.

The overall pattern of inhibition by infant saliva was neither “mouse-like” nor “ferret-like” (Figure 2C). Similar to the mouse, human saliva neutralized Udorn to a significantly lower level than PR8 (*p* < 0.05), and inhibition of Udorn was largely RDE-sensitive (*p* < 0.05). However, unlike the mouse, inhibition of PR8 was also mainly RDE-sensitive (*p* < 0.01). This finding is in agreement with previous reports in the literature showing that the major anti-influenza activity of saliva is associated with sialic-acid-containing molecules [18]. However, when the inhibitory properties of each infant sample were examined individually (Figure 3), it was clear for certain individuals (I2, M1, N1, O1, F1, and P1) that sialic acid-independent inhibition formed the major component of the anti-influenza activity of their saliva (Figure 3A). Of the 17 saliva samples where both the RDE-treated and untreated could be tested, only D1 showed a classical “mouse-like” profile of inhibition while F1, P1, and possibly O1 were “ferret-like”. The majority of the samples tested displayed a statistically significant decrease in the inhibition of both PR8 and Udorn after RDE treatment (E1, Q1, A1, B1, and G1) or of just PR8 (L1, S1, C1, H1, and R1) (Figure 3B). Inhibition by a number of these latter samples was lower for the RDE-treated than untreated saliva against Udorn, but this difference did not reach statistical significance. Nevertheless, the level of neutralization of Udorn by RDE-treated saliva for those samples in Figure 3B was significantly less than by the untreated saliva (*p* < 0.01). 

The residual RDE-resistant inhibition by the infant saliva samples against Udorn could be mediated by SP-D, MBP, and SAP, which act as classic β inhibitors, binding to mannose-rich glycans on the viral HA to sterically block access of the HA to cell surface receptors. However, PR8 is neutralized poorly, if at all, by SP-D and MBP due to the lack of carbohydrate side chains on the PR8 HA head [34]. Furthermore, Hartshorn et al. [5] previously found that SP-D contributed little to the anti-IAV activity of saliva and that some of the antiviral activities of SP-D were antagonized by salivary gp-340 [19]. The residual RDE-resistant inhibition of PR8 is therefore more likely due to SAP or an inhibitor analogous to the “mouse-like” neuraminidase inhibitor that is highly active against PR8 [23]. The observation that the concentration of specific salivary proteins can differ among individuals has been previously noted [17]. Differential expression levels of specific antiviral proteins could partly explain why viruses like influenza can infect some individuals more efficiently than they do others.

### 3.3. Inhibitors in Infant Saliva Differed with Age But Not Gender

**Figure 4:**
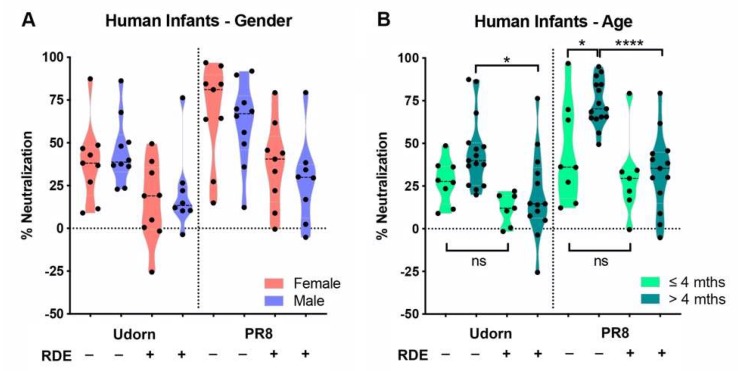
Effects of infant gender and age on inhibition of IAV by human saliva. PR8 or Udorn virus (5000 PFU) were added to untreated or RDE-treated saliva from (**A**) male or female infants or (**B**) infants of different ages (≤ or >4 months) and the percent neutralization determined as described in Figure 2. Violin plots depicting the mean of each group and probability density of the data are shown and represent the results from individual human infant saliva samples tested at least in triplicate.

An analysis of gender- and age-associated differences in inhibition across the different groups was then performed by comparing the neutralization of each strain by the saliva of females and males, and by saliva taken from infants ≤4 months of age compared to those >4 months. The results showed that there was no gender bias in the pattern of salivary inhibition (Figure 4A). However, in terms of age, a small but significant difference (*p* < 0.05) was noted in the neutralization of PR8 by the untreated saliva of infants ≤4 months of age compared to those >4 months (Figure 4B). In addition, while the inhibition of PR8 by untreated saliva was significantly greater than by the RDE-treated saliva of infants >4 months (*p* < 0.0001), which mirrored the inhibition profile of the entire saliva panel (*p* < 0.001, Figure 2C), this difference was not apparent in the saliva of infants ≤4 months (Figure 4B). This implies that infants ≤4 months of age have a much lower abundance of sialic-acid-bearing inhibitors in saliva compared to older infants. Notably, the three highest responders in the ≤4 month-untreated group were D1 (16.4 weeks of age, 96.8% inhibition of PR8), S1 (16.1 weeks, 69.8%), and C1 (18.0 weeks, 63.8%) all right on the 4-month cut-off used for the analysis. Restricting the analysis to less than four months (effectively removing these three samples) did not leave sufficient remaining samples to provide statistical power; however, this observation suggests that very young infants clearly lack or have a greatly reduced component of inhibition in saliva mediated by sialic acid-containing inhibitors. While the results for Udorn showed that there was no age-associated difference in inhibition by untreated saliva, a similar trend was observed to that noted above. Namely, the small difference observed against Udorn between untreated vs. RDE-treated saliva in the entire saliva panel (*p* < 0.05, Figure 2C) was maintained in the saliva of infants >4 months (*p* < 0.05, Figure 4B), but was lost in the saliva of infants ≤4 months, which also implies that younger infants have a diminished RDE-sensitive (sialic-acid-mediated) salivary component of inhibition. These data are consistent with MUC5B, a known influenza inhibitor [16], as being responsible for or contributing to the sialic acid-dependent inhibition. Unlike other salivary components, expression levels of this mucin are low for the first five months of infancy, but become more abundant toward the end of the first year [35].

**Figure 5:**
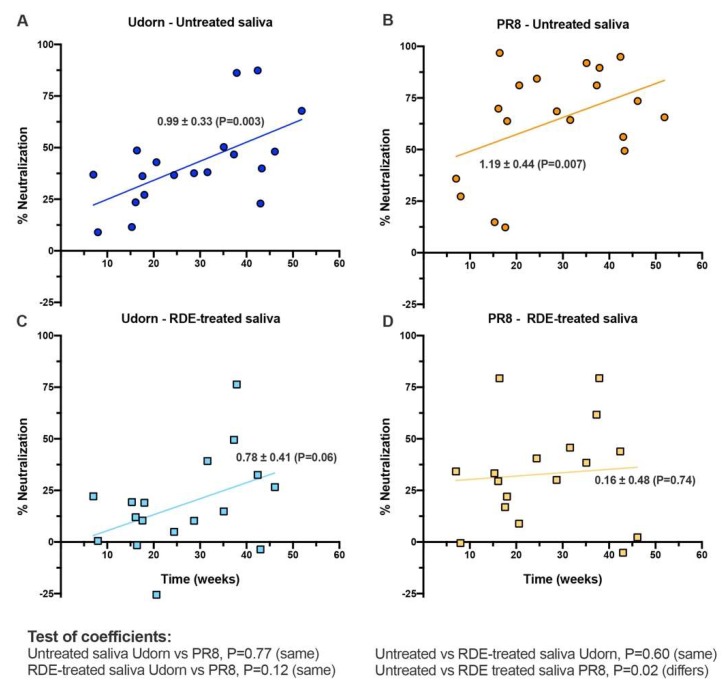
Correlation of infant age with neutralization of IAV by linear regression. Data shows linear regression analysis performed for (**A**,**B**) untreated or (**C**,**D**) RDE-treated saliva against (**A**,**C**) Udorn or (**B**,**D**) PR8 virus. Data-points represent the means of 19 untreated and 17 RDE-treated individual human infant saliva samples tested at least in triplicate. Indicated for each line of best fit are the regression coefficients ± standard error (*P* value indicative of the goodness of fit of data to the line). Below the figure, the results of pairwise tests of the similarity of the slopes of the lines are indicated.

To confirm an association with age, independent regression analyses were performed on the entire saliva panel. The ability of untreated saliva to neutralize both Udorn and PR8 viruses increased significantly over the first year of life (Figure 5A,B). The ability of RDE-treated samples to neutralize Udorn showed the same relationship with age, but a suppressed level of activity overall when compared to untreated saliva (Figure 5C). RDE treatment of saliva increased the heterogeneity of responses against PR8 and removed the previously observed association with age (Figure 5D). These analyses confirmed that the overall abundance or activity of inhibitory molecules active against IAV increased with age in infants, however, the sialic acid-independent inhibitor component active against PR8 was present at lower and varied levels in individuals, independent of age. Importantly, saliva from even the youngest donors in our study showed some capacity to inhibit influenza.

### 3.4. Inhibition of the 2009 Pandemic H1N1 Virus by Infant Saliva Is Less Than That of PR8 

**Figure 6:**
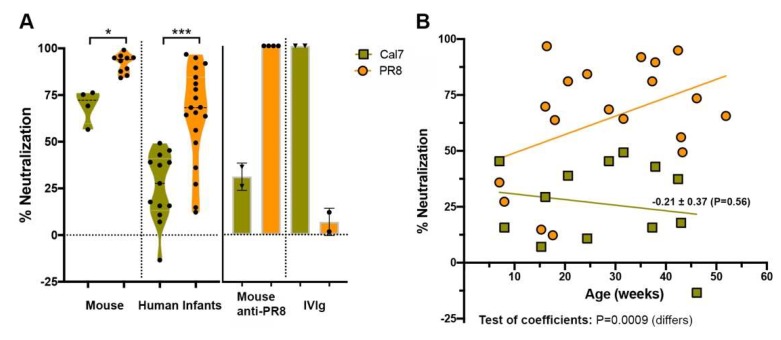
Inhibition of H1N1 IAV by the saliva of human infants. (**A**) A/California/07/2009 (Cal7) or PR8 virus (5000 PFU) were added to untreated mouse or human infant saliva and the percentage neutralized calculated as described for Figure 2. Inhibition by PR8 hyper-immune mouse sera or intravenous immunoglobulin (IVIg) prepared from 2013 human blood donors (0.05 mg/mL) were included as controls (*n* = 2–4 replicate samples). Violin plots depicting the mean of each group and probability density of the data at different values are shown and represent the results of 4–10 independent experiments from pooled mouse saliva or at least triplicate samples from 13–19 individual human infants. (**B**) Linear regression analysis performed on untreated saliva against PR8 and A/California/07/2009 viruses. Data-points represent the means of 13–19 untreated individual human infant saliva samples tested at least in triplicate. Indicated for the line of best fit for Cal7 are the regression coefficient ± standard error (*P* value indicative of the goodness of fit of data to the line). Below the figure, the results of tests for similarity of the slopes of the regression lines for PR8 (Figure 5) and Cal7 are indicated.

To further explore the inhibitory response, neutralization of the recent pandemic H1N1 A/California/7/2009 strain was investigated. Inhibition of this virus by both untreated mouse saliva (*p* < 0.05) and untreated human infant saliva (*p* < 0.001) was lower than that of the historical H1N1 PR8 virus (Figure 6A). The specificity of plaque inhibition was verified using anti-PR8 hyperimmune sera raised in mice and intravenous immunoglobulin (IVIg) prepared from 2013 human blood donors containing IgG antibodies.

Regression analysis showed that unlike for PR8, where the inhibitory activity of untreated saliva increased over time, inhibitory activity remained uniformly low against A/California/7/2009 (Figure 6B). This observation agreed with similar findings by Limsuwat et al. [18,31], who showed a comparatively low inhibition of pandemic H1N1 by human saliva relative to that of seasonal H1N1 strains. Chen et al. [20] demonstrated that A2M and an A2M-like protein were essential components of salivary innate immunity against a related pandemic H1N1 isolate. The observation that untreated infant saliva neutralized the A/California/7/2009 virus relatively poorly and that activity remained uniformly low across the age distribution suggests that infant saliva might not contain high levels of A2M or A2M-like proteins.

## 4. Conclusions

Although some studies have investigated age- and sex-associated differences in the glycopatterns of human salivary glycoproteins [25,26,27,28] and their roles against the influenza virus [29], the youngest of these cohorts have been three years of age. Here, we show that even very young infants have some protection against IAV from innate inhibitors, in large part from those acting as sialic acid-containing decoys, which increase over the first year of life. We also saw a lower and more variable activity that was sialic acid independent in its function. Finally, our results showed that the pattern of activity of individual infants could be quite different, which is reflective of the presence of different inhibitory mediators that may shape the effectiveness of their earliest responses to the virus.

## Appendix A

**Table A1:** Infant saliva samples.

Infant ID	Age (weeks)	Gender
I1	6.1	Male
I2^1^	7.0	Male
R1	8.0	Female
N1	15.3	Female
S1	16.1	Male
D1	16.4	Female
M1	17.6	Male
C1	18.0	Female
E1	20.6	Female
A1	24.4	Female
L1	28.7	Male
S2	31.1	Male
O1	31.6	Female
B1	35.1	Male
F1	37.3	Female
S3	37.6	Male
P1	37.9	Male
G1	42.4	Female
Q1	43.0	Male
J1	43.3	Male
A2	44.1	Female
H1	46.1	Male
K1	51.9	Male

^1^ Indicates second sample from infant I.
